# Multicenter randomized study on the comparison between electronic and traditional chest drainage systems

**DOI:** 10.1186/s13063-019-3811-8

**Published:** 2019-12-16

**Authors:** Giuseppe Marulli, Giovanni M. Comacchio, Mario Nosotti, Lorenzo Rosso, Paolo Mendogni, Giuseppe Natale, Luigi Andriolo, Giovanna Imbriglio, Valentina Larocca, Debora Brascia, Federico Rea

**Affiliations:** 1 Thoracic Surgery Unit, Department of Organ Transplantation and Emergency, University Hospital of Bari, Piazza Giulio Cesare, 11, 70124 Bari, Italy; 2 Thoracic Surgery Unit, Department of Cardiologic, Thoracic and Vascular Sciences, University Hospital of Padova, Padova, Italy; 3 Thoracic Surgery Unit, Fondazione IRCCS Ca’ Granda Ospedale Maggiore Policlinico, University of Milan, Milan, Italy; 4 Thoracic Surgery Unit, ‘V Fazzi’ Hospital, Lecce, Italy

**Keywords:** Traditional chest drainage systems, Digital chest drainage, Air leakage, Chest tube removal, Lobectomy

## Abstract

**Background:**

In patients submitted to major pulmonary resection, the postoperative length of stay is mainly influenced by the duration of air leaks and chest tube removal. The measurement of air leaks largely relies on traditional chest drainage systems which are prone to subjective interpretation. Difficulty in differentiating between active air leaks and bubbles due to a pleural space effect may also lead to tentative drain clamping and prolonged time for chest drain removal. New digital systems allow continuous monitoring of air leaks, identifying subtle leakage that may be not visible during daily patient evaluation. Moreover, an objective assessment of air leaks may lead to a reduced interobserver variability and to an optimized timing for chest tube removal.

**Methods:**

This study is a prospective randomized, interventional, multicenter trial designed to compare an electronic chest drainage system (Drentech™ Palm Evo) with a traditional system (Drentech™ Compact) in a cohort of patients undergoing pulmonary lobectomy through a standard three-port video-assisted thoracic surgery approach for both benign and malignant disease. The study will enroll 382 patients in three Italian centers. The duration of chest drainage and the length of hospital stay will be evaluated in the two groups. Moreover, the study will evaluate whether the use of a digital chest system compared with a traditional system reduces the interobserver variability. Finally, it will evaluate whether the digital drain system may help in distinguishing an active air leak from a pleural space effect, by the digital assessment of intrapleural differential pressure, and in identifying potential predictors of prolonged air leaks.

**Discussion:**

To date, few studies have been performed to evaluate the clinical impact of digital drainage systems. The proposed prospective randomized trial will provide new knowledge to this research area by investigating and comparing the difference between digital and traditional chest drain systems. In particular, the objectives of this project are to evaluate the feasibility and usefulness of digital chest drainages and to provide new tools to identify patients at higher risk of developing prolonged air leaks.

**Trial registration:**

ClinicalTrials.gov, NCT03536130. Retrospectively registered on 24 May 2018.

## Background

Length of stay (LOS) impacts significantly on health-care costs. In patients submitted to major pulmonary resection, the LOS is mainly influenced by the duration of air leaks and chest tube removal, which are the main factors that delay hospital discharge. Prolonged air leaks affect between 10 and 15% of pulmonary surgical procedures and may be a risk factor for increased morbidity, prolonged hospital stay and increased costs [1, 2]. The measurement or grading of air leaks still relies on a static analog measurement of “bubbles in a chamber” using traditional chest drainage systems. These systems are inherently prone to subjective interpretation and observer variability conditioned by habit and personal clinical experience [3]. Moreover, the difficulty in differentiating between an active air leak and bubbles due to a pleural space effect, which are indistinguishable on checking a traditional water-seal chest drain system, may lead to tentative drain clamping and prolonged time for chest drain removal.

The introduction of novel digital chest drainage systems could give specific advantages.

First of all, they could give an objective assessment of real air leak, reduce interobserver variability and, finally, optimize the timing of chest tube removal. Continuous monitoring of air leaks may identify subtle leakage that may not be visible during daily patient evaluation. Moreover, these systems could distinguish an active air leak from an apparent air leak due to a pleural space effect through the evaluation of differential intrapleural pressures [4]. Finally, these systems could identify patients at higher risk of prolonged air leak and this could be useful in prompting either early active intervention or conversion to a one-way valve system that would allow for outpatient management and therefore early discharge from the hospital [5]. Some of these systems feature portable suction systems, allowing earlier mobilization of the patients, thus reducing complications due to bed immobility. To date, few studies (mostly single-center studies with small numbers of patients) have been performed with the aim to evaluate the clinical impact of digital drainage systems [5, 6]. The works published on this subject were performed using different electronic devices: some of them use an air flow meter to directly measure the airflow through the chest tube, whereas others derive these data from an algorithm based on the intrapleural pressure maintained by a suction pump and measured through a pressure sensor [5, 7, 8].

These previous studies proved that electronic drainage systems contribute to early mobilization, shorten the duration of chest tube placement, and thus hospital stay, and reduce costs using a more accurate and objective air leak assessment. Moreover, these systems are more effective in standardizing postoperative management of chest tubes [6, 7]. These advantages have particular importance in patients undergoing pulmonary resection with an underlying pulmonary disease such as COPD [9]. Finally, another possible advantage is to avoid tentative clamping since digital drainage eliminates subjective estimation. Brunelli et al. [5] described the possibility of predicting the risk of prolonged air leak after pulmonary lobectomy through the analysis of both air leak flow and pleural pressure. Such analysis cannot obviously be performed through a standard water-seal chest drain system.

All of these studies underlined the potential clinical utility and impact of electronic drainage systems; however, prospective randomized studies with a large number of patients are needed to corroborate these results.

Therefore, we intend to perform a trial to compare electronic chest drainage system performances to those of a traditional system in a prospective cohort of patients who received thoracoscopic lobectomy. Both systems are currently used in our daily clinical practice.

### Primary outcomes

- To determine whether the use of a digital chest system compared with a traditional system reduces the duration of chest drainage and LOS

#### Primary outcome measures

To determine whether the use of a digital chest system compared with a traditional system reduces the duration of chest drainage and LOS, and to evaluate the role of interobserver variability, the duration of chest tube stay, the hospital length of stay and the daily presence of active air leaks will be evaluated in the two groups.

### Secondary outcomes

- To quantify the variability of results regarding the subjective observer evaluation of active air leaks (through the traditional system) compared with the objective data registered by the digital system
- To distinguish an active air leak from a pleural space effect by the evaluation of intrapleural differential pressure
- To identify potential predictors of prolonged air leaks

#### Secondary outcome measures

To distinguish an active air leak from a pleural space effect and to identify potential predictors of prolonged air leaks, the digital chest system will record and evaluate the intrapleural differential pressure, the flow of air leaks and the daily variation of these two parameters in each patient.

## Methods/design

This study is designed as a prospective randomized, multicenter, investigator-initiated study, of an interventional type, performed at three high-volume Italian thoracic surgery units: Thoracic Surgery Unit of Padua University Hospital, Thoracic Surgery Unit of Fondazione IRCCS Cà Granda Ospedale Maggiore Policlinico— University of Milan and Thoracic Surgery Unit of Vito Fazzi Hospital, Lecce.

**Fig. 1:**
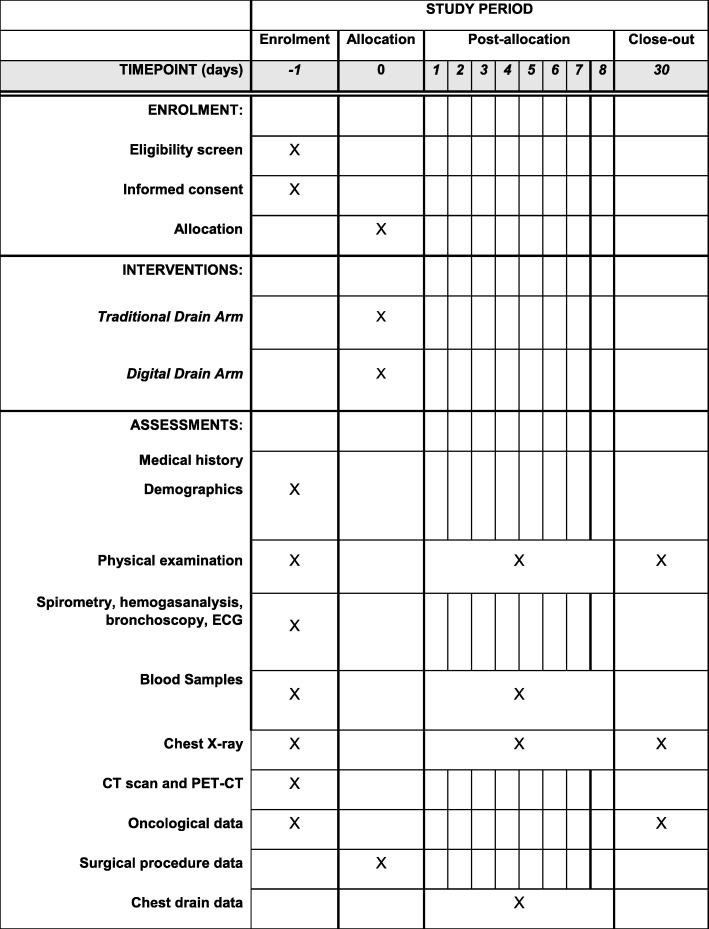
Standard Protocol Items: Recommendations for Interventional Trials (SPIRIT) figure for the schedule of enrollment, interventions and assessments. CT computed tomography, ECG electrocardiogram, PET positron emission tomography

**Fig. 2:**
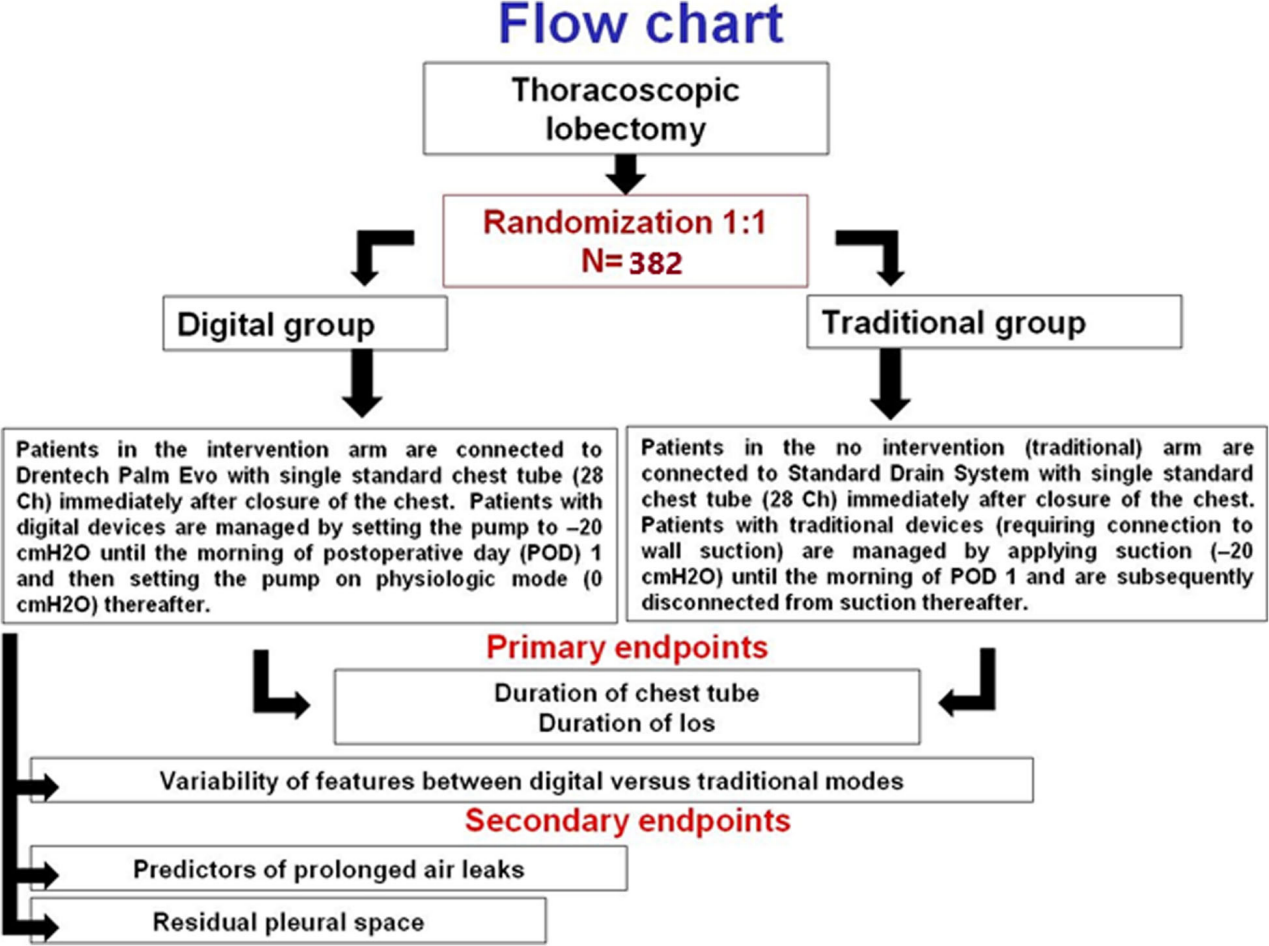
Trial flow chart. los length of stay

This protocol is reported in line with the Standard Protocol Items: Recommendations for Interventional Trials (SPIRIT) guidelines using the SPIRIT figure (Fig. 1), trial flow chart (Fig. 2) and SPIRIT Checklist (Additional file 1).

The study will enroll 382 patients undergoing pulmonary lobectomy through a standard uniportal, biportal or triportal VATS approach, either for benign or malignant disease.

The sample size was calculated considering a two-sided *t* test for a difference in means outcome, according to the following assumptions: effect size = 0.33 for both primary endpoints, power (1 – β) = 0.8 and α = 0.025 accounting for a Bonferroni adjustment (α = 0.05 / 2) [10]. The sample size computation was performed with R software [11].

Potential participants will be identified and recruited into the trial by surgeons who work in the aforementioned thoracic surgery units.

Patients will be enrolled if they meet the inclusion criteria specified afterward and sign the informed consent form (ICF).

An interim analysis will be performed on the primary endpoint when 200 patients are enrolled and randomized.

### Inclusion criteria

- Able and willing to read, understand and provide written informed consent
- Patients undergoing VATS lobectomy
- Age 18–80 years
- Gender: both
- Estimated life expectancy of at least 6 months.
- Tumor considered potentially resectable by R0 surgery
- Adequate respiratory function for surgery, particularly a predicted postoperative (ppo) forced expiratory volume in 1 s (FEV1) greater than 30% and a ppo carbon monoxide lung diffusion capacity (DLCO) greater than 30%, in addition to maximum oxygen consumption greater than 10 ml/kg/min
- Must have signed and dated an ICF before performance of any study-specific procedures or tests—subjects must be fully informed about their illness and the investigational nature of the study protocol

### Exclusion criteria

- Patients requiring ICU care with mechanical ventilation
- Patients needing reintervention during postoperative care
- Patients requiring a thoracotomy
- Tumor considered potentially resectable by incomplete surgical resection with microscopic residual disease (R1) or gross residual disease (R2)
- Evidence of extra-thoracic disease
- Major thoracic surgical procedure before enrollment
- Any other significant comorbid condition that, in opinion of the investigator, would impair study participation or cooperation

### Screening

Subjects are screened prior to the surgery. The following activities and/or assessments will be performed during screening:

- Obtain written ICF from subject or subject’s legal representative
- Collect subject’s medical history
- Record concomitant medications
- Perform physical examination
- Record vital signs, height and weight
- Collect blood sample for hematology, coagulation tests, blood chemistry, renal and liver function tests and measurement of electrolytes (obtain within 30 days prior to the surgery)
- Record 12-lead electrocardiogram (ECG)
- Record spirometry, hemogasanalysis and bronchoscopy
- Perform baseline CT scan, PET–CT scan and chest X-ray
- Perform anesthesiologic evaluation

### Device description

The Drentech™ Palm EVO device is a portable vacuum unit connected to a compatible collection system.

The collection system is a standard disposable drainage unit that can be connected to a chest drain, with a water-seal valve.

The unit is capable of generating suction that can be independently adjusted by a central vacuum and/or energy sources. The unit can be powered and/or the batteries recharged using the power supply provided.

The mobile unit is equipped with a display to show the following information:

- Indication of the “instantaneous” air leakage corresponding to the last minute of operation
- Mean air leakage value of the patient in the last hour of measurement
- Indication of the hours of device operation, calculated starting from the moment of activation
- History of the air leakages and minimum and maximum intrapleural pressures relating to the last 99 h of operation, available in numerical and graphic form
- Measurement and storage of the minimum and maximum intrapleural pressure values of the patient relating to the last minute of operation
- Real-time measurement of the minimum and maximum intrapleural pressure values for each respiratory act of the patient

The unit can transfer data to a USB storage device in order to view and/or store them on a PC. The USB storage device can be connected to the dedicated port on the unit using the adapter provided, and the data can be viewed with standard spreadsheet programs or the RedaxPlot software provided.

### Randomization

A person not involved with either enrollment, assessments or training of participants will generate the allocation sequence. Individual randomization, stratified for centers and with blocking to reduce variability, will be performed with a 1:1 allocation to the intervention and control groups. The assignment to one of the two devices will be performed using closed envelopes containing notes reading either “T” for traditional water seal system or “D” for digital system. The randomization will be done in the surgical theater at the end of thoracoscopic lobectomy by one of the surgeons by opening the envelope assigned to the patient. Due to the nature of the intervention, neither participants nor investigators will be masked during the intervention.

### Intervention

The day of the surgery, after induction of anesthesia, a double-lumen endotracheal tube is used for selective ventilation of the lungs. During the procedure, patients are monitored by ECG, arterial line, pulse oximeter and urine output. Patients are placed in a lateral decubitus and undergo a VATS lobectomy associated with lymphadenectomy. During surgery, the patient receives fluid and, eventually, blood replacement, maintaining adequate blood pressure and urine volume. After VATS lobectomy, the presence of air leaks is tested by a water submersion test under standard airway pressure of 25 cm H_2_O and the air leaks are measured by a volumetric system. In the case of significant air leaks (i.e. more than 100 ml/min), application of sutures is allowed, whereas no buttressing material, sealants or pleural tents are permitted. At the end of the procedure, a single apical 28-Ch chest tube is placed.

Patients are then randomized 1:1 to receive two different types of chest drainage management: digital, connected to the Drentech™ Palm Evo system; or traditional, connected to the water-seal drain system already in use in each center. After surgery, patients are transferred to the ward.

Patients with the digital chest drainage system are managed by setting the pump to − 20 cmH_2_O from the moment the patient is extubated until the morning of the first postoperative day (POD) and then turning off the suction (0 cmH_2_O). Patients with traditional devices are connected to wall suction (− 20 cmH_2_O) until POD 1, and then they are disconnected from suction.

The following activities and/or assessments will be performed after surgery:

- ▪ Daily assessment of the cardio-pulmonary parameters (blood pressure, cardiac frequency, oxygen saturation) all through the hospitalization
- ▪ Complete blood count, liver and renal functional tests the first day after surgery and then depending on the clinical condition of the patient or medical judgment
- ▪ Chest X-ray immediately after the return to the ward and afterward depending on clinical condition of the patient or medical judgment; after drains are removed, another chest X-ray is performed before discharge

Postoperative treatments include respiratory rehabilitation and mobilization, and antithrombotic and antibiotic prophylaxis. Pain will be controlled by means of analgesic drugs according to each center’s analgesic protocol based on its routine practice (intravenous, epidural, intercostal block or mixed regimens).

The chest tube is removed when chest X-rays show a complete lung expansion and there is no detectable air leak on traditional devices. For digital devices, absence of air leaks is defined as a recorded airflow lower than 20 ml/min, with suction set at 0 cmH_2_O, for at least 8 h and without significant spikes of air leak on the graph. The daily fluid drainage threshold for drain removal is based on each center’s routine practice (generally 300 ml). During morning and evening rounds, the presence of an air leak and the pleural effusion volume are checked for both types of drain system. In particular, the Drentech™ Palm Evo is equipped both with the standard traditional “water-seal” system and with the digital electronic device: for the evaluation of correspondence between subjective clinical observation of air leaks and objective digital results, the presence of air leaks will be checked on each visit round by two clinicians via observation of bubbles in the chamber with the digital screen switched off in order to be blinded regarding the digital features. Then, the electronic screen is switched on and the data are recorded. For the purpose of clinical management, in this group of patients, the clinician responsible for the ward will consider only the features of the digital device.

In the case of a “pleural space” effect suspected on the basis of features of the digital system, a provocative clamping maneuver will be performed and a control chest X-ray at 24 h will be done for confirmation to decide on chest drain removal. Air leak duration is calculated, as usual, from the day of operation until the day an air leak is no longer detectable. Duration of air leaks for more than 7 days are considered prolonged air leaks and are managed according to each center’s routine (e.g. discharge with a portable chest drainage system or reoperation). Moreover, in these cases, the air leak duration will be measured and data will be collected until the 8th POD and no discharge from the hospital will be considered before that day.

### Follow-up

Postoperative follow-up will consist of a scheduled visit with chest X-ray about 30 days after discharge.

### Study termination and drop out

The patient has the right to terminate the research and evaluation at any time point, without sacrificing further medical treatment. There is no replacement of patients who withdraw from the study or who interrupt prematurely. The reason for discontinuation of the study will be given on the data collection form.

### Statistical analysis

Periodical checking of the input data will be performed in order to verify the completeness and consistency of the database.

Checks on the consistency and plausibility of the reported data will be carried out prior to data analysis.

The study is powered based on its primary endpoints: the duration of chest tube placement and the length of stay. The sample size was calculated to detect a difference in duration of chest tube placement and/or hospital stay after thoracoscopic lobectomy of at least 1 day and based on previously published data (standard deviation = 3) [5]. A sample size of 382 patients (191 patients per group) was determined based on 90% statistical power, with a significance level of 0.05, and allowing for dropouts.

### Risks and benefits for the patient

The patient’s participation in the study will not involve additional risks other than those related to normal clinical and surgical practice for pulmonary lobectomy. The complications occurring after operation until discharge, or within 30 days post operation in discharged patients, are considered postoperative complications; death during the same period is defined as perioperative death. Postoperative complications are described according to the Common Terminology Criteria for Adverse Events 4.1 (CTCAE version 4.1) published by the National Cancer Institute of the USA, reported, classified and recorded in case report form (CRF).

### Data collection and management

The CRF of the study will be paper based (Additional file 2). Data will be collected at the time of patient enrollment in the study, during hospitalization and at 1 month after discharge.

All data collected will be recorded in a computer database (Microsoft Excel) with the protection of a password. A sequential identification number will be attributed to each patient registered in the study. This number will identify the patient and must be included on all case report forms.

The database will be kept at the Thoracic Surgery Unit of Padua University Hospital. The data will be collected during the preoperative assessment, during surgery, in the postoperative days during the daily evaluation of patients (at two visit rounds, in the morning and in the afternoon) and at 1 month after discharge from the hospital.

## Discussion

Among patients undergoing pulmonary resection, the postoperative management of chest tubes remains a critical issue. No standardized guidelines are available and, particularly for air leakage assessment, tube management conventionally depends on the experience of individual surgeons; therefore, interobserver disagreement is frequent [1, 2]. Finally, an apparent air leakage may be related to a pleural space effect and, in such cases, a clamping trial should be performed to rule out the existence of small occult air leaks before removal [4].

To date, few studies have been performed to evaluate the clinical impact of digital drainage systems [5–9, 12]. Previous published studies proved that electronic drainage systems reduce the interobserver variability, allowing earlier removal of chest tubes and reducing the length of hospital stay and costs [5, 11]. Brunelli et al. [5], in fact, proved a decrease in LOS of 0.9 days (5.4 vs 6.3 days) when comparing digital vs traditional systems, allowing for a decrease in postoperative costs of €476 per patient (€2391 vs €2867). Furthermore, Cerfolio and Bryant [11] showed a decrease in LOS of 0.7 days (3.3 days vs 4.0 days) when comparing digital vs traditional systems, with a mean day for chest tube removal of 3.1 vs 3.9 in favor of the digital system. These studies underline the potential clinical utility and impact of electronic drainage systems; however, they have been performed using different electronic devices—some of them use an air flow meter to directly measure the airflow through the chest tube, whereas others derive these data from an algorithm based on the intrapleural pressure maintained by a suction pump and measured through a pressure sensor.

The proposed trial will provide new knowledge to this research area by investigating and comparing the difference between digital and traditional chest drain systems, evaluating not only clinical outcomes but also more theoretical issues that influence clinical practice.

Indeed, our aim is to determine not only whether the use of a digital chest system compared with a traditional system reduces the duration of chest drainage and LOS as shown by the previous studies, but also to quantify the variability of results regarding the subjective observer evaluation of active air leaks (through the traditional system) compared with the objective data registered by the digital system by evaluating the daily presence of active air leaks.

Another endpoint of our study is to identify any predictive factor of prolonged air leaks; however, these findings will need further studies for their clinical validation.

Finally, among the patients enrolled in the digital system group, we aim to explore the potentiality for continuous monitoring of the intrapleural differential pressure, the flow of air leak and the daily variation of these two parameters, allowing us to distinguish an active air leak from a pleural space effect by the evaluation of intrapleural differential pressure and to identify potential predictors of prolonged air leaks.

The results of this project will provide novel information about the feasibility and usefulness of the digital chest drain and can potentially provide new tools to identify patients at higher risk of developing prolonged air leaks.

## Trial status

The study started in April 2017. The first patient was enrolled on April 5, 2017. The study will continue until sufficient power is reached, approximately until March 2020. Protocol version n. 3, February 15, 2017.

## Supplementary information

**Additional file 1.** SPIRIT 2013 Checklist: Recommended items to address in a clinical trial protocol and related documents.

**Additional file 2.** Case report form (CRF).

## Data Availability

Not applicable.

## Abbreviations

COPD: Chronic obstructive pulmonary disease
CRF: Case report form
CT: Computed tomography
ICF: Informed consent form
ICU: Intensive care unit
LOS: Length of stay
POD: Postoperative day
VATS: Video-assisted thoracic surgery
