# Supplementary material for: Multicenter randomized study on the comparison between electronic and traditional chest drainage systems
Source: Trials. 2019 Dec 16;20:730. doi: 10.1186/s13063-019-3811-8 (PMC6915964; doi:10.1186/s13063-019-3811-8)
Supplement: Supplementary file 2 — Additional file 2. Case report form (CRF). [file 13063_2019_3811_MOESM2_ESM.doc]

|  | **VARIABLE** | **DESCRIPTION** | |
| --- | --- | --- | --- |
|  | PATIENT ID |  | |
|  | SEX | 1. M 2. F | |
|  | DATE OF BIRTH |  | |
|  | AGE |  | |
|  | COMORBIDITIES | 1. No 2. Yes | |
| **PATIENT DATA** | CORONARY ARTERY DISEASE | 1. No 2. Yes | |
|  | BLOOD HYPERTENSION | 1. No 2. Yes | |
|  | VALVE DISEASE | 1. No 2. Yes | |
|  | STROKE OR TIA | 1. No 2. Yes | |
|  | DEMENTIA | 1. No 2. Yes | |
|  | PERIPHERAL VASCULAR DISEASE | 1. No 2. Yes | |
|  | COPD | 1. No 2. Yes | |
|  | DIABETES | 1. No 2. Yes | |
|  | ASTHMA | 1. No 2. Yes | |
|  | OTHER NEOPLASMS | 1. No 2. Yes | |
|  | FEV1 |  | |
|  | FEV1% |  | |
|  | FVC |  | |
|  | FVC% |  | |
| **PRE-OPERATIVE TESTS** | TLC |  | |
|  | TLC% |  | |
|  | RV |  | |
|  | RV% |  | |
|  | Tiffenau |  | |
|  | DLCO% |  | |
|  | EGA (NORMAL RANGE) | 1. No 2. Yes | |
|  | TC | TNM | |
|  | PET FDG | 1. Positive  2. Negative | |
|  | FBS (PATHOLOGICAL FINDINGS) | 1. No 2. Yes | |
| **ONCOLOGICAL DATA** | PREOEPRATIVE HISTOLOGICAL DIAGNOSIS | 1. Adk 2. Squamous cell 3. Carcinoid 4. Lymphoma 5. Metastasis 6. SCLC 7. other 8. No | |
|  | NEOADJUVANT CHEMOTHERAPY | 1. No 2. Yes | |
|  | NEOADJUVANT RADIOTHERAPY | 1. No 2. Yes | |
|  | GRAY |  | |
|  | DATE OF SURGERY |  | |
| **SURGICAL DATA** | LOBECTOMY | 1. RUL 2. ML 3. RLL 4. LUL 5. LLL | |
|  | DURATION (MIN) |  | |
|  | LYMPHADENECTOMY | 1. Radical 2. Sampling 3. Not done | |
|  | AIR LEAKS AT END OF SURGERY (ml/min) |  | |
|  | HISTOLOGY | 1. Adk 2. Squamous cell 3. Carcinoid 4. Lymphoma 5. Metastasis 6. SCLC 7. Other | |
| **POSTOPERATIVE HISTOLOGICAL DIAGNOSIS** | T |  | |
|  | N |  | |
|  | M |  | |
|  | GRADING |  | |
| **RANDOMIZATION** |  | 1. Digital 2. Traditional | |
| **POST OEPRATIVE DATA (FOR EVERY POST-OPERATIVE DAY TILL THE EIGHTH)** | **DAY N.** | | |
|  | AIR LEAKS (MORNING) | 1. No  2. Yes | ml/min  (only for digital device) |
|  | AIR LEAKS (AFTERNOON) | 1. No 2. Yes | ml/min  (only for digital device) |
|  | DIGITAL DEVICE / WATER SEAL AGREEMENT | 1. No 2. Yes |  |
|  | PLEURAL FLUID LOSS (ml / 24h) |  | |
|  | CHEST X-RAY | 1. Not done 2. Normal 3. Pneumothorax 4. Parenchymal thickening 5. Pleural effusion | |
|  | RESIDUAL PLEURAL SPACE | 1. No 2. Yes | |
|  | CLAMPING | 1. No 2. Yes | |
|  | DRAIN REMOVAL | 1. No 2. Yes | |
|  | POST OPERATIVE MORBIDITY | 1. No 2. Yes | |
|  | CARDIOVASCULAR | 1. Atrial fibrillation 2. AMI 3. Stroke, TIA | |
| **GENERAL POST OPERATIVE DATA** | PULMONARY | 1. Prolonged air leaks (>7gg) 2. Pulmonary embolism 3. ARDS 4. Pneumonia 5. Atelectasis 6. Residual pleural space 7. Sputum retention | |
|  | SURGICAL | 1. Hemothorax 2. Bronchial fistula 3. Chylothorax 4. Phrenic nerve palsy | |
|  | KIDNEY | 1. Acute renal failure 2. Urinary tract infection | |
|  | GASTROINTESTINAL | 1. Acute abdomen 2. Diarrhea | |
|  | OTHER |  | |
|  | DEATH | 1. No 2. Yes | |
|  | DAY OF DISCHARGE |  | |
